# Low Levels of Procalcitonin or Presepsin Combined with Significantly Elevated C-reactive Protein May Suggest an Invasive Fungal Infection in Hematological Patients With Febrile Neutropenia

**DOI:** 10.1097/HS9.0000000000000170

**Published:** 2019-01-08

**Authors:** Igor Stoma, Igor Karpov, Anatoly Uss, Svetlana Krivenko, Igor Iskrov, Natalia Milanovich, Svetlana Vlasenkova, Irina Lendina, Kristina Belyavskaya, Veranika Charniak

**Affiliations:** 1 Belarusian State Medical University, Department of Infectious Diseases, Minsk, Belarus; 2 Minsk Scientific Practical Center of Surgery, Transplantation and Hematology, Belarus

Infectious complications in immunocompromised patients with neutropenia or on long-lasting immunosuppressive treatments are a serious issue. Invasive fungal infections (IFIs) represent cases in which delayed or inappropriately targeted treatments can have fatal consequences. Today, *Aspergillus spp.* and *Candida spp.* are the 2 most important genera accounting for about 95% of all cases of IFIs.^1^ The early diagnosis of IFI still remains a complicated issue nowadays.^2,3^ In combination with clinical and imaging data, detection of galactomannan antigen in aspergillosis and mannan antigen in candidemia is broadly used. However, concentration of these antigens is related to the invasiveness of the pathogens.^4–6^ PCR assays are considered to be promising detection methods, but not yet recommended for routine use in clinical practice because of the lack of conclusive validation for commercially available assays, variety of methodologies in the literature, and questions about the extent to which results assist diagnosis.^7^ Owing to the diagnostic issues and complicated detection of fungi, diagnosis of IFIs is still expressed on a scale of probability: proven, probable, and possible.^7,8^ Based on current recommendations from European Conference on Infections in Leukemia and Guidelines from Infectious Diseases Society of America, in case of febrile neutropenia, empirical antifungal treatment is prescribed after 72 to 96 h of broad-spectrum antibacterial treatment without an observed clinical improvement.^9,10^ It means that in a large number of cases, patients with neutropenia and fungal infection do not receive any effective therapy at least for the first 72 h of fever episode, what may have an expected effect on risk of a fatal outcome. Owing to the above-mentioned practical issues, there is a need to distinguish between bacterial and fungal infections as early as possible in patients with fever and neutropenia. Sepsis biomarkers are among the broadly implemented diagnostic methods nowadays, with C-reactive protein (CRP), presepsin, and procalcitonin (PCT) being most frequently used.

Although levels of CRP as an acute phase protein are elevated in many inflammatory conditions and used to monitor inflammation in many fields of medicine, increase in PCT is associated with bacterial infections.^11^ Presepsin (sCD14), a novel biomarker implemented in clinical practice in 2004, is a receptor of lipopolysaccharide–lipopolysaccharide-binding protein complexes which is generated as the body response to bacterial infection, taking into account that phagocytosis plays a major role in immune response to bacteria.^12^ Furthermore, there is a lack of compelling information concerning the use of presepsin in hematological patients, and there is a practical need to assess the diagnostic characteristics of combinations of biomarkers in hematological patients. Therefore, as a basis for this study, we have taken the clinical experience at our tertiary hematology and bone marrow transplantation center, where we have observed a number of cases of discordant results of increased CRP and low levels of PCT or presepsin in patients with subsequently confirmed IFIs.

Here, we describe the results of a prospective observational clinical study that was performed in our tertiary hematology and bone marrow transplantation center during the period from 2013 to 2018. The study was approved by the Institutional Review Board and Ethics Committee. Adult patients hospitalized to receive chemotherapy for hematological malignancies or treatment of graft-versus-host disease after allogeneic hematopoietic stem cell transplantation with an episode of microbiologically proven bacterial/fungal infection were included in the study. Patients with febrile episodes were screened for infections by means of standard hospital protocols, and during the first 48 h after onset of the fever biomarkers (CRP combined with PCT or presepsin) were measured. Either PCT or presepsin were measured in all patients based on physicians’ decision, obligatory during the first 48 h after fever onset and non-obligatory later, depending on clinical course of the patient. Onset of the febrile episode in all of the patients was during the hospitalization period. Blood samples (for microbiological analysis and biomarker detection) were taken before the initiation of empiric antibacterial therapy, and all of the biomarkers were measured in fresh plasma. Clinical diagnosis of IFIs was based on probability classification.^13^ Galactomannan antigen blood test was performed during the first 96 h of febrile episode, with a blood culture gathered in all cases during the first 24 h. CT scan was routinely performed in high-risk patients with febrile neutropenia. Fungal diagnostic tests included blood cultures for *Candida spp*. and sputum cultures confirmed by galactomannan antigen in blood for *Aspergillus spp*. Diagnosis of bloodstream infection (BSI) was made according to CDC criteria, whereas all included patients were divided into a group with either bacterial or fungal infection. The study was conducted in accordance with STARD 2015 guidelines and according to Helsinki declaration. Analysis of diagnostic parameters of combination of biomarkers was performed using a logistic regression model with 2 biomarkers as the explanatory variables, and subsequent receiver operating characteristic curve (ROC) analysis of predicted probabilities from that model.

**Figure 1:**
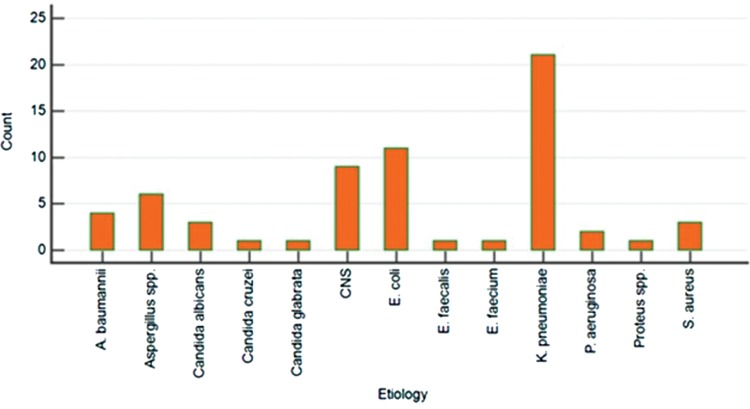
**Spectrum of infectious episodes among patients in the study.**

**Figure 2:**
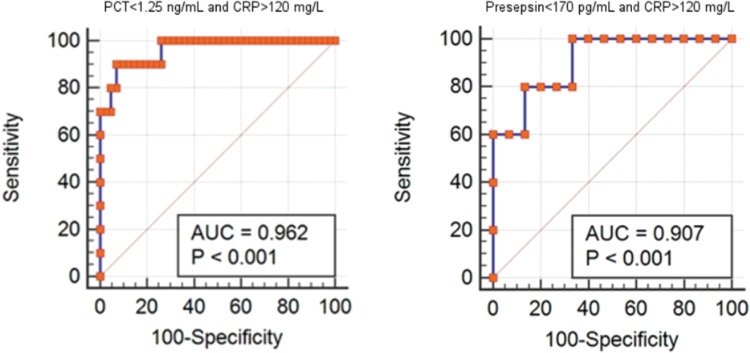
**ROC-curves for combinations of sepsis biomarkers in diagnosis of IFIs in hematological patients.** IFI = invasive fungal infections.

The total number of patients with hematological neoplasms seen in the described unit during the study period was 1246 patients, including 218 patients with fulfilled criteria of febrile neutropenia. Among them, 64 patients had an episode of microbiologically proven bacterial/fungal infection. Therefore, the microbiology was positive in 29.4% of patients with febrile neutropenia. The median age of included patients was 41 years (interq. int. 34–51); 34 (54%) were male. Primary diagnoses included acute myeloid leukemia (42/66.7%), acute lymphoblastic leukemia (6/9.4%), multiple myeloma (5/7.8%), and other hematological diseases, whereas 48 (75%) of patients had ANC below 500 cells/mm^3^ at the onset of a febrile episode. Infectious episodes had a bacterial origin in 53 (82.8%) and fungal origin in 11 (17.2%) cases (Fig. 1). During the preliminary analysis, we have already observed that in most of the patients with IFIs, there were low numbers of PCT or presepsin and high numbers of CRP, what corresponded with our clinical expectations. As a first step of ROC analysis, we have performed the estimation of optimal cut-off for IFIs in all of studied biomarkers based on Youden index, with optimal cut-off values assessed as follows: >120.4 mg/L for CRP; ≤1.26 ng/mL for PCT, and ≤173 pg/mL for presepsin. Based on this findings, we have performed the second step of analysis estimating the diagnostic characteristics of combinations “low PCT and high CRP” and “low presepsin and high CRP” with the above-mentioned cut-off values. Expectedly, both of the combinations showed high-quality diagnostic parameters, what is shown in Table 1 and on ROC curves in Figure 2.

**Table 1:**
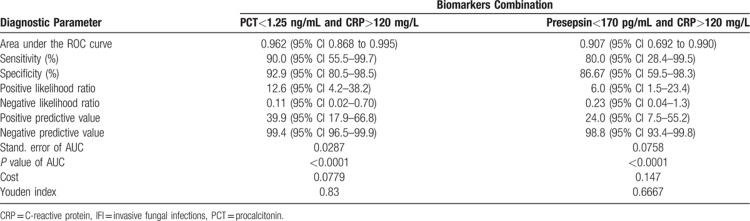
Diagnostic parameters of combinations of CRP, PCT and presepsin as indicators of IFIs in hematological patients

To our knowledge, this is the only study that focused on clinical significance of combinations of presepsin, CRP, and PCT for early diagnosis of IFI and guided empirical antifungal treatment. There is a published research paper on combination of biomarkers for diagnostics of fungal infections in patients with chemotherapy-induced immunosuppression, wherein Marková et al on a smaller cohort of hematological patients have shown the phenomenon of “low PCT and high CRP” in case of fungal infections.^14^ There were also data published on CRP and PCT use in surgical patients in ICU, wherein increase in CRP in addition to normal PCT showed the shift of predictive effect from bacterial infections toward candidemia.^15^ It is also interesting to mention that elevation of CRP was observed in most of the patients in our study, whereas the elevation of PCT or presepsin was found only in patients with bacterial BSIs. The low practical value of measuring PCT as a marker of fungal infections was reported earlier by other groups, whereas there are no data published yet on presepsin use in patients with fungal infections.^16–18^ One of the practical issues of the observed results is the fact that CRP is an easily accessible and obtainable biomarker to test in most of clinical settings, whereas specific fungal biomarkers (1,3-beta-D-glucan, galactomannan) and PCR-based diagnostic methods are less accessible for regular monitoring in hematology centers. Therefore, based on the observed results, the combinations of sepsis biomarkers (ie, seriously elevated CRP, whereas PCT or presepsin remain in low concentrations) may be used as a possible method to distinguish between bacterial and fungal infections when microbiology/histology results are pending. This may lead to an earlier start of empirical antifungal treatment in hematology.
